# Fat cadherins in mouse models of degenerative ataxias

**DOI:** 10.1038/s41598-019-52684-7

**Published:** 2019-11-06

**Authors:** Olga Baron, Denise Grieshober, Catarina Dias, Manolis Fanto

**Affiliations:** 1 Department of Basic and Clinical Neuroscience, King’s College London, 125 Coldharbour Lane, SE5 9NU London, United Kingdom; 2 Wolfson Centre for Age-Related Disorders, King’s College London, Guy’s Campus, SE1 1UL London, United Kingdom; 3 Institut du Cerveau et de la Moelle épinière (ICM), 47, bd de l’hôpital, F-75013 Paris, France

**Keywords:** Spinocerebellar ataxia, Gene expression

## Abstract

Autophagy is a lysosomal degradation pathway that plays an essential role in neuronal homeostasis and is perturbed in many neurological diseases. Transcriptional downregulation of *fat* was previously observed in a *Drosophila* model of the polyglutamine disease Dentatorubral-pallidoluysian atrophy (DRPLA) and this was shown to be partially responsible for autophagy defects and neurodegeneration. However, it is still unclear whether a downregulation of mammalian *Fat* orthologues is associated with neurodegeneration in mice. We hereby show that all four *Fat* orthologues are transcriptionally downregulated in the cerebellum in a mouse model of DRPLA. To elucidate the possible roles of single *Fat* genes, this study concentrates on *Fat3*. This *fat* homologue is shown to be the most widely expressed in the brain. Conditional knockout (KO) of Fat3 in brains of adult mice was attempted using the inducible Thy1Cre(ER^T2^) SLICK H line. Behavioral and biochemical analysis revealed that mice with conditional KO of *Fat3* in the brain display no abnormalities. This may be ascribed either to the limited efficiency of the KO strategy pursued or to the lack of effect of *Fat3* KO on autophagy.

## Introduction

Fat cadherins regulate growth, mitochondria and planar cell polarity during development in mammals and *Drosophila*^1^. In *Drosophila* some of these events are regulated through the transcriptional repressor Atrophin^2^ and through the Hippo pathway^3^. In mammals, there are four *fat*-related cadherins: Fat1, Fat2, Fat3 and Fat4. Compared to *Drosophila*, the regulation of the Hippo pathway by the four Fat homologues is less well understood in mammals and some of the links identified in *Drosophila* appear to be missing. Recently, however, it was found that Fat1 directly interacts with Mst1, one of the mammalian Hippo kinases, in head and neck squamous cell carcinoma leading to association of a multimeric signalling complex. This complex enables phosphorylation of Mst1 by the thousand-and-one amino acid kinases (TAOKs) and therefore activation of the Hippo pathway^4^. In addition, Fat4 was found to indirectly regulate Yap1 in the mouse heart by regulating nuclear translocation of the Yap1 partner Angiomotin-like 1 (Amotl1) but by skipping the Hippo core complex^5^.

Apart from modulating cell proliferation and apoptosis, the signalling by Fat cadherins and Hippo kinases regulate autophagy, a catabolic pathway of extreme relevance to tumour growth, cellular homeostasis and cell death^6^. Several non-exclusive mechanisms were identified in *Drosophila* and mammals though which the Fat and Hippo pathway regulate autophagy^7–11^. Autophagy has an essential neuroprotective role and autophagy defects are observed in many neurodegenerative diseases^12^.

The polyglutamine (polyQ) disorders are a recognised cause of inherited neurodegeneration and are characterized by misfolding and intraneuronal accumulation of mutated polyQ proteins. The autosomal dominant ataxia Dentatorubral-pallidoluysian atrophy (DRPLA) is caused by CAG triplet expansion mutation in the *ATROPHIN-1* (*ATN-1*) gene^13^. Patients with expansion between 49 and 65 CAG repeats display atrophy of the brainstem and cerebellum associated with symptoms such as ataxic gait, choreoathetosis and dementia^14^. In *Drosophila* models of DRPLA based on the expression in photoreceptor neurons of either a mutant human ATN-1 with a 65Q expansion or of an engineered *Drosophila* Atrophin with an equivalent (75Q) expansion^15^, the Fat/Hippo pathway is downregulated, resulting in exacerbation of neurodegeneration through autophagy disorders^10^. Atrophin directly regulates *fat* as a transcriptional target^10,16^ and, mutants in *fat* or *hippo* pathway core components show a built-up of autophagic vesicles in their photoreceptors indicating a block in the autophagy flux^10^.

A mouse model for DRPLA, based on transgenic expression throughout the brain with the PrP promoter of a mutant human ATN-1 encoding for a 65Q expansion closely recapitulates the late-onset DRPLA with predominantly cerebellar degeneration, ataxic gait, anxiety and premature death^17^. In this model, we have recently reported a thorough characterisation of autophagy defects most prominent in the cerebellum^18^, indicating similarities with the *Drosophila* model.

Here, we set out to establish whether the link between DRPLA, Fat and neurodegeneration is conserved in mammals. We show that, in the mouse model used for DRPLA all four mammalian Fat homologues are significantly downregulated in the cerebellum, the most affected brain area in this model. *Fat3* is most widely expressed in the mouse brain, however a partially efficient conditional pan-neuronal deletion of *Fat3* is not sufficient to cause neurodegeneration and autophagy defects.

## Results

### All four *Fat* orthologues are transcriptionally downregulated in the cerebellum of DRPLA mice

**Figure 1:**
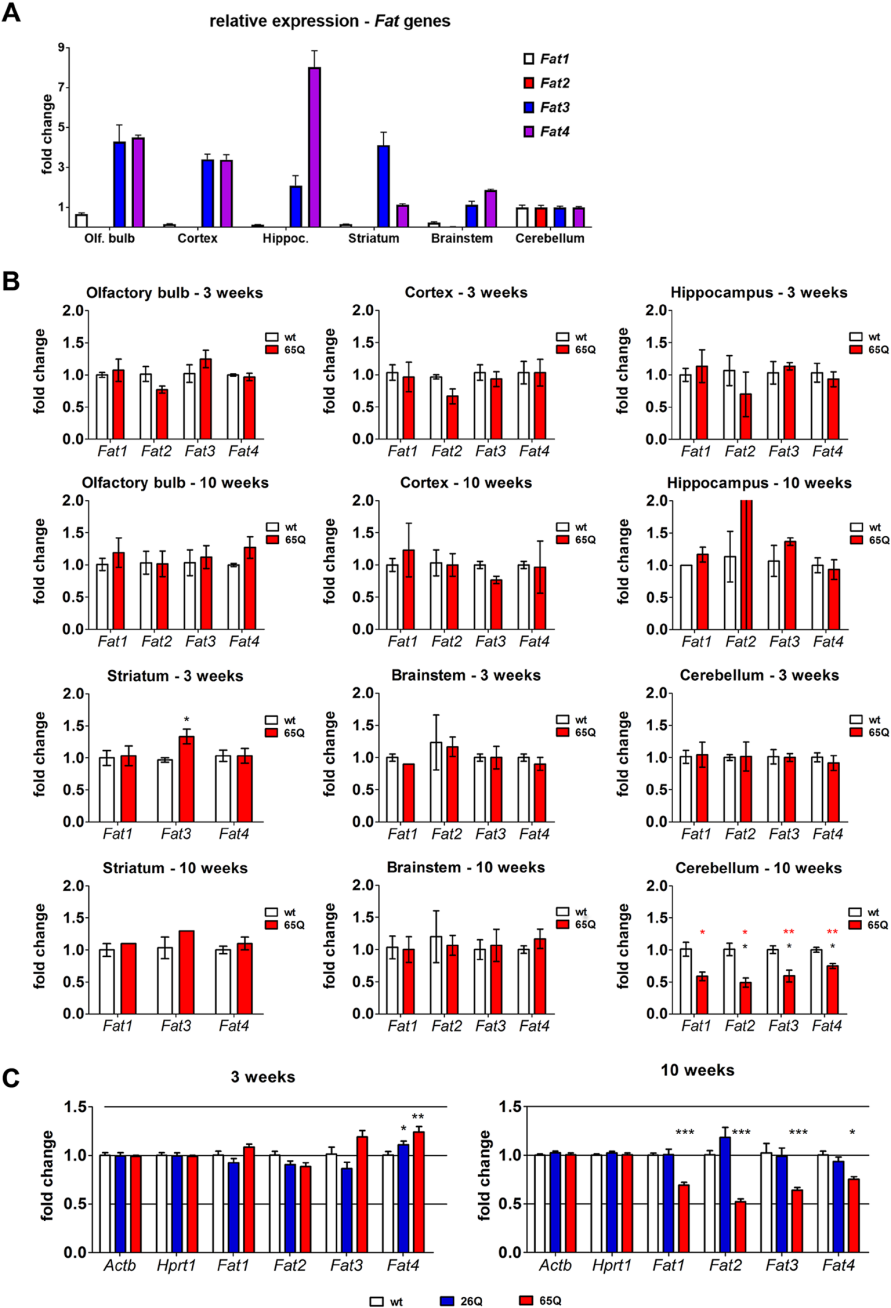
Specific downregulation of *Fat* gene expression in the cerebellum of the early symptomatic mice (10 weeks). (**A**) Relative mRNA levels of *Fat1* (white), *Fat2* (red), *Fat3* (blue) and *Fat4* (purple) in wild type mice at 10 weeks (red) in the C57BL/6J; C3H background. The levels were determined by qPCR normalised against *Hprt1* as a housekeeping gene. Fold change for Olfactory bulb (Olf. bulb), Hippocampus (Hippoc), Striatum, Brainstem and Cortex was normalised to Cerebellum tissue. Graph represents mean values ± SEM (n = 3 animals). (**B**) Relative mRNA levels of *Fat1*, *Fat2*, *Fat3* and *Fat4* in wild type mice (wt; white) and ATN1-FL-65Q mice (65Q; red) at 3 and 10 weeks of age in cerebellum, brainstem, striatum, hippocampus, cortex, and olfactory bulb in strains kept on a (CBA/Ca × C57BL/6J) F1 background. The levels were determined by qPCR normalised against *Hprt1* as a housekeeping gene. Fold change is given as mean ± SEM relative to wild type (n = 3 animals). DataAssist™ software (Thermo Fisher Scientific) *p < 0.05 and **p < 0.01 with (black) or without (red) FDR correction. (**C**) qPCR analysis of mRNA levels of 4 mammalian *Fat* paralogues in the cerebellum of the presymptomatic 3 week (left) and 10 week (right) old wild type (wt, white), ATN1-FL-26Q (blue) and ATN1-FL-65Q mice (red). Relative levels normalised to *β-actin* and *Hprt1* are given as a fold change of wild type; Two-way ANOVA, n = 6, mean ± SEM, ***p < 0.001, **p < 0.01, *p < 0.05.

Previous work on the Drosophila DRPLA models highlighted the role of direct transcriptional regulation of the *fat* gene encoding for the gigantic fat cadherin as an early onset Atrophin specific molecular pathomechanism, which is partly responsible for the neurodegeneration and the autophagy defects^10^. In the mammalian brain all four *Fat* orthologues were reported to be expressed in a spatially differential manner and *Fat3* was generally shown to be the highest expressed in the brain^19^. Therefore, we performed an initial qPCR analysis of the four mammalian *Fat* orthologues in several brain areas. The relative *Fat* gene expression was unchanged between 3- and 10-weeks old mice, however, the spatial abundance relative to the cortex levels of the different *Fat* genes was very different. Hereby, *Fat2* was almost exclusively present in the cerebellum, while *Fat1* showed a stronger expression in the olfactory bulb and cerebellum. *Fat3* and *Fat4* showed a rather ubiquitous expression (Fig. 1A).

The comparative analysis between wt and DRPLA (ATN1-FL-65Q) mouse lines revealed a significant downregulation of all *Fat* paralogues in the cerebellum of ATN1-FL-65Q mice at the early symptomatic time point of 10 weeks but not at 3 weeks (Fig. 1B). Also, no differences were observed at any stage in any of the other brain regions analysed (Fig. 1B), despite the fact that the Prnp promoter used for the generation of the DRPLA mouse models is ubiquitously active in the brain^20^. A more highly powered analysis, including a control line for wt Atrophin overexpression (ATN1-FL-26Q) confirmed significant downregulation of all Fat paralogues in the cerebellum, specifically for the pathological ATN1-FL-65Q strain at 10 weeks of age (Fig. 1C). The regional specificity strongly correlates with previous observation of stalled canonical autophagy and neurodegeneration in the cerebellum of DRPLA mice^18^. These results suggest a striking conservation of the transcriptional downregulation of *fat* identified in Drosophila^10^ and further indicate that downregulation of *Fat* might correlate with the pathological mechanism in DRPLA patients.

### Conditional pan-neuronal induction of recombination by Thy1CreER^T2^

**Figure 2:**
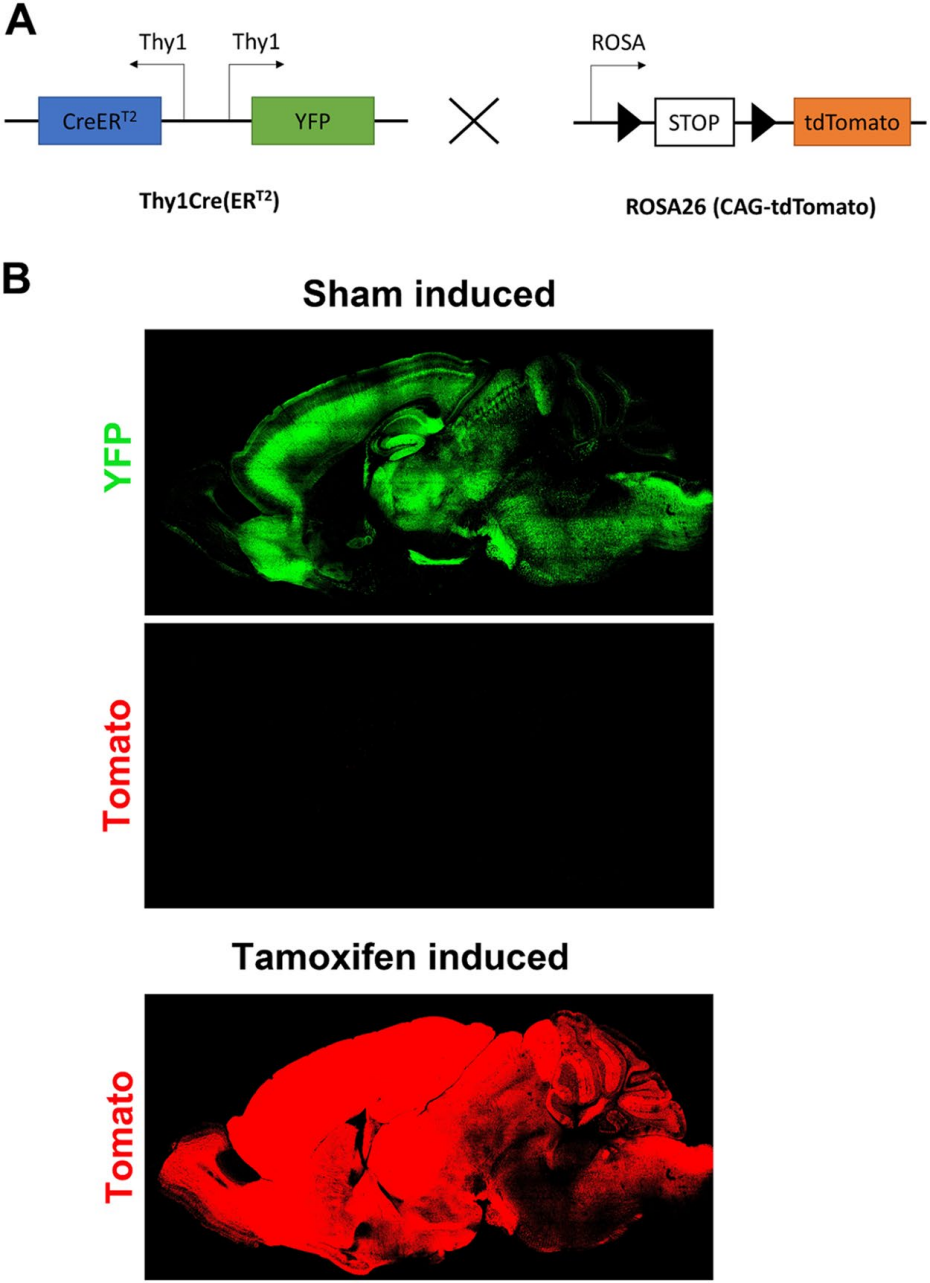
Conditional induction of Thy1Cre(ER^T2^) works. (**A**) The SLICK-H recombination system was tested prior to this study by breeding SLICK-H mice with a B6.Cg-Gt(ROSA)26Sor < tm14(CAG-tdTomato)Hze>/J reporter line. The SLICK-H line expresses both the CreER^T2^ transgene and YFP under the control of two copies of the Thy1 promotor. Tamoxifen dependent translocation of CreER^T2^ into the nucleus leads to recombination of flanking LoxP sides, excision of NeoR Stop cassette sand subsequent expression of tdTomato. (**B**) Representative brains of control mice mice which were sham injected with corn oil (upper) and mice which were injected with tamoxifen (lower). No tdTomato expression can be observed in control mice when tamoxifen is absent. In induced mice, tdTomato is highly expressed in the forebrain and the brainstem and more weakly in the cerebellum.

To test whether mutations in one of the Fat paralogues could elicit age related neurodegeneration and autophagy defects specifically in the neurons, an approach would be required to bypass developmental requirement for Fat cadherins in the nervous system^21^ and to allow other organs to rely on Fat cadherins functions throughout adult life. To this aim, we set out to use the SLICK-H Cre recombination system. This system expresses both the CreER^T2^ transgene and YFP under the control of two copies of the Thy1 promotor and therefore enables pan-neuronal, tamoxifen-induced deletion of a gene of interest^22^. As “leaky” recombination in the absence of tamoxifen administration was observed in some CreERT2 transgenic lines^23^, the recombinase activity was tested in presence and absence of tamoxifen prior to this study by breeding SLICK-H mice with a CAG-tdTomato reporter line (Fig. 2A). The absence of red fluorescence in the sham-induced mice with corn oil only confirmed that the line is not leaky in the absence of tamoxifen. The conditional induction of Thy1Cre(ER^T2^)-mediated genomic recombination was effective throughout the brain allowing targeting of several areas, however, it appeared somewhat more efficient in the forebrain compared to the cerebellum (Fig. 2B) as reported previously^23^.

### Efficiency of tamoxifen-induced *Fat3* deletion in different brain areas

**Figure 3:**
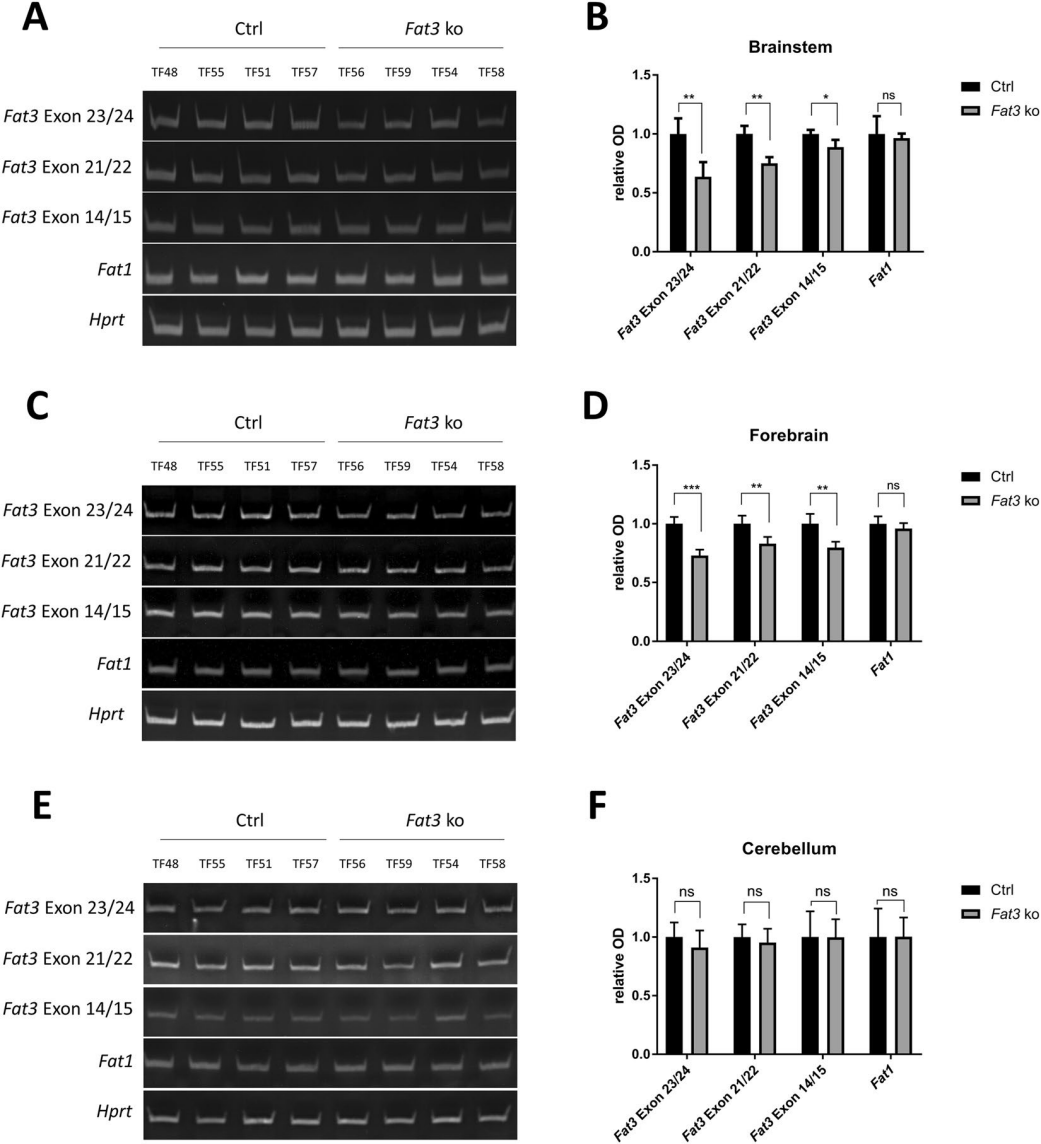
Semi-quantitative analysis of *Fat3* mRNA levels in brainstem, forebrain and cerebellum. *Fat3* mRNA levels are partially downregulated in brainstem and forebrain of Fat3 deficient mice but not altered in the cerebellum. Semi-quantitative analysis of *Fat3* RNA level was performed for brainstem (**A**,**B**) forebrain (**C**,**D**) and cerebellum (**E**,**F**). *Hprt1* and *Fat1* were used as loading controls. Values were normalized to *Hprt1* and are given as fold change relative to control. Student’s t-test, mean ± SD, n = 4 per group, *p ≤ 0.05, **p ≤ 0.01, ***p ≤ 0.001.

*Fat3* was reported to be the most widely expressed in many regions in the brain^19,24,25^ and therefore less likely to be compensated in brain areas with limited expression of other Fat homologues, which are mostly restricted to cerebellum and olfactory bulb. The expression pattern of *Fat3* is therefore most suitable to targeting through the SLICK-H line, which is more active outside the cerebellum. We therefore decided to focus on this *Fat* gene paralogue and to use a floxed Fat3 allele, previously generated to elucidate the function of Fat3 in neuronal morphology^26^. Cre-induced recombination of this allele deletes exon 23 in the Fat3 gene and was reported to eliminate Fat3 protein expression upon successful recombination. The use of the tamoxifen-inducible SLICK-H line assures correct neuronal development to proceed and allows us to focus on primary neurodegenerative events triggered in the adult nervous system by Fat3 deficiency. Given the absence of detectable leakiness from the SLICK-H line, siblings *Fat3*^*f/fl*^ missing *Thy1Cre*(*ER*^*T2*^) and injected with tamoxifen were chosen as control mice to account for possible effects of this drug. To test the efficiency of *Fat3* deletion in the brain of these mice, we assessed the of level *Fat3* mRNA in different brain areas of *Fat3* ko mice. Using semi-quantitative PCR, and different exon-spanning primer pairs we analysed the presence of the deleted exon 23 as well as non-deleted exons 21/22 and 14/15. Densitometric analysis of the PCR products reveals that exon 23 is reduced by 30 to 40% in the brainstem and the forebrain (Fig. 3A–D). Levels of exons 21/22 were decreased by 20 to 30% and exons 14/15 were reduced by only 10 to 20%. To corroborate the specificity of this reduction in *Fat3* mRNA levels we assessed the *Fat1* mRNA levels remained unchanged in any brain area analysed. Compared to brainstem and forebrain, *Fat3* mRNA levels are not significantly altered in the cerebellum (Fig. 3E,F). In conclusion, *Fat3* is shown to be reduced in the brainstem and forebrain and not altered in the cerebellum.

### Fat3 conditional KO mice do not display any overt neurological phenotype

**Figure 4:**
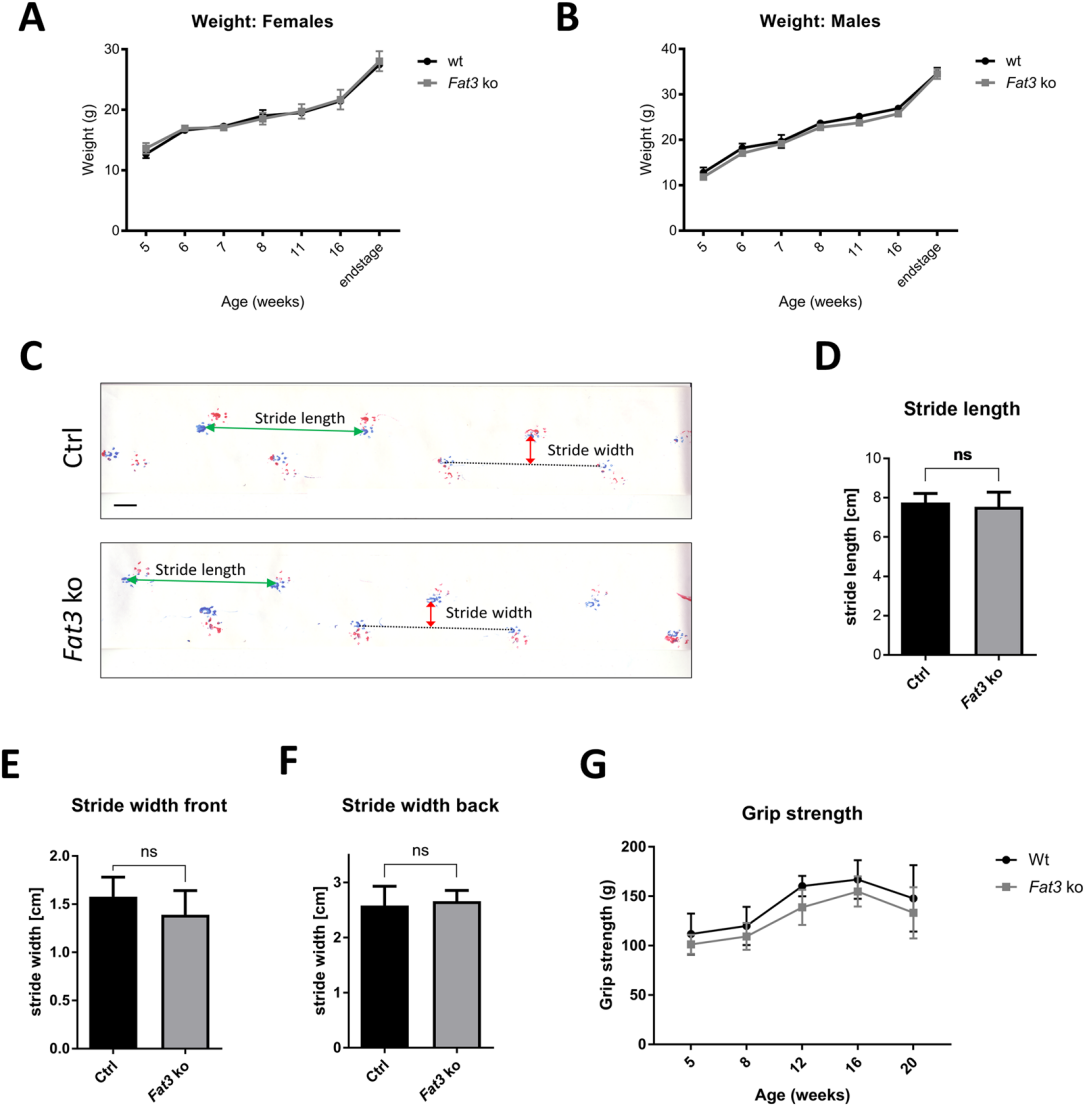
Fat3 KO mice display no behavioural abnormalities. (**A**,**B**) Body weight was assessed every week starting at five weeks of age and at endstage. Both females (**A**) and males (**B**) showed no abnormalities in body weight at any time point analyzed. Repeated measures two-way ANOVA was performed to test for statistical significance. Each value is given as mean ± SEM. (**C**–**F**) Gait analysis was performed at 19 months of age. (**C**) Representative footsteps of control and Fat3 ko mice. Front limbs are coloured blue and hind limbs are coloured red, respectively. This image was digitally manipulated to remove a registered trademark. Scale bar 1 cm. Stride length (**D**), front step width (**E**) and back step width (**F**) were determined for 4 animals per group (n = 4). Student’s t-test, mean ± SD. (**G**) Grip strength of neuronal Fat3 ko mice is mostly normal compared to control mice. Grip strength was analyzed over time (once a week) and repeated measures two-way ANOVA was performed to test for statistical significance. Values are given as mean ± SEM.

Having established that Fat3 was partially deleted in brainstem and forebrain, we examined whether this reduction was sufficient to generate behavioural defects. *Fat3* ko mice generated were, however, normal in appearance and general behaviour and in body weight up to 19 months of age (Fig. 4A,B). Gait analysis revealed no abnormalities between mice with or without *Thy1Cre*(*ER*^*T2*^) construct. No significant changes were found in stride length (Fig. 4D) as well as front (Fig. 4E) and back (Fig. 4F) step width. Grip strength test was initially performed to evaluate neuromuscular function and was shown to be unchanged between the two groups (Fig. 4G). No obvious motor anomalies, grid suspension inability or limb clasping were visually detected up to 19 months, when mice were sacrificed. In conclusion, mice with partial neuronal *Fat3* depletion do not show any overt behavioural abnormalities that would indicate neurodegeneration.

### Mice with neuronal deficiency for *Fat3* display unchanged autophagic flux

**Figure 5:**
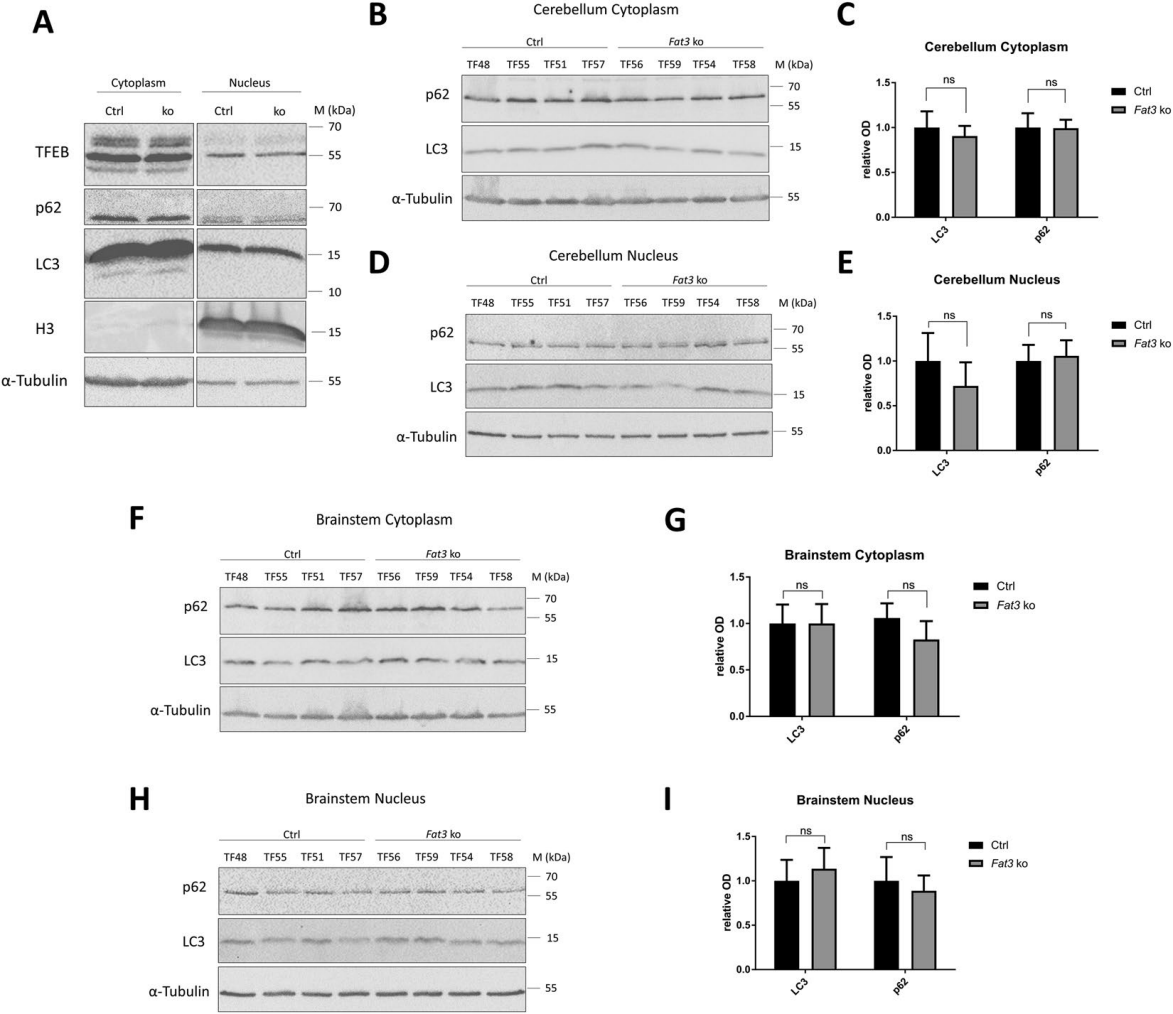
Fat3 KO mice display unchanged LC3 and p62 levels in the cerebellum and brainstem. (**A**) Western blot analysis of cerebellar lysate fractions shows an accumulation of TFEB, p62, LC3 and α-Tubulin in the supernatant while H3 is enriched in the pellet. The LC3 antibody detects a doublet consisting of LC3I (upper) and a faint band for LC3II (lower). 50 µg of total protein was loaded for the supernatant and 25 µg for the pellet. (**B**) Cerebellar cytoplasmic fraction. 50 µg total protein were loaded per sample. (**C**) Quantitative analysis of LC3 and p62 levels in the cerebellar cytoplasmic fraction. (**D**) Cerebellar nuclear fraction. 25 µg total protein were loaded per sample. (**E**) Quantitative analysis of LC3 and p62 levels in the cerebellar nuclear fraction. (**F**) Brainstem cytoplasmic fraction. 50 µg total protein was loaded per sample. (**G**) Quantitative analysis of LC3 and p62 levels in the brainstem cytoplasmic fraction. (**H**) Brainstem nuclear fraction. 25 µg total protein were loaded per sample. (**I**) Quantitative analysis of LC3 and p62 levels in the brainstem nuclear fraction. The LC3 antibody detects only one band corresponding to LC3I. α-Tubulin was used as a loading control. Student’s t-test, mean ± SD, n = 4 per group.

Autophagy defects were previously observed in photoreceptor neurons of *Drosophila* mutants for *fat*^10^, we therefore sought to establish whether *Fat3* deficient mice show similar defects. Characterisation of protein lysate fractions obtained upon brain tissue homogenisation from 19 months aged animals showed an enrichment in transcription factor EB (TFEB), phosphorylated TFEB (pTFEB, upper band), p62, LC3I, a faint LC3II band and α-Tubulin in the supernatant. Histone 3 (H3) instead accumulates in the pellet (Fig. 5A). In conclusion, the supernatant is enriched in cytoplasmic proteins while the pellet is enriched in nuclear proteins. Therefore, they are further referred to as cytoplasmic and nuclear enriched fractions.

To test for possible alterations in autophagic flux, LC3 and p62 levels were determined using western blot analysis of brain lysates from control and *Fat3* ko mice. Given the differential efficiency detected in *Fat3* transcript downregulation in the different brain areas, we have compared the markers for autophagy flux in separated cerebellar and brainstem lysates. In agreement with the lack of detected *Fat3* depletion in the cerebellum, there was no alteration of LC3I and p62 levels in this area in both cytoplasmic and nuclear enriched fractions (Fig. 5B–E). Surprisingly, however, LC3I and p62 levels were also normal in the brainstem of *Fat3* deficient mice (Fig. 5F–I), i.e. in a part of the brain that had shown a significant downregulation in *Fat3* mRNA. The LC3II band was hardly detected in any of the lysates, evidencing no accumulation of this autophagosome marker. This result, together with the absence of p62 accumulation, suggests that at a global level there is no evidence of impairment or alteration in the autophagic flux in mouse brain with a partial reduction in *Fat3* expression.

## Discussion

Although SLICK-H was reported to have high recombination efficiencies among NeuN positive cells in the brainstem and many areas in the forebrain, Cre recombination efficiencies were previously observed to be lower in the cerebellum^23^. Here, we obtained similar results by breeding SLICK-H mice with the CAG-tdTomato reporter line which led to a weaker expression of tdTomato in the cerebellum compared to forebrain and brainstem (Fig. 2B). The reduced recombination efficiency in the cerebellum may explain the complete absence of a significant reduction of *Fat3* mRNA levels in *Fat3* ko mice in this brain area (Fig. 3E–F) as well as the lack of autophagy disorders in the cerebellum (Fig. 5B–E). To target specifically the cerebellum, it may therefore be preferable to use alternative Cre lines to SLICK-H, for instance the generated *Nse*-*CreER*^*T2*^ ^27^ that targets specifically granule cells, in which most Fat orthologues are highly expressed.

Although a high recombination efficiency was expected in the forebrain and the brainstem, qPCR shows a reduction of the deleted exon 23 of *Fat3* by only 30 to 40% (and of the non-deleted exon 14/15 by only 10–20%) in those areas (Fig. 3A–D). This result may reflect largely the fact that mRNA was extracted from whole brain areas and therefore includes a mixture of many different cell types. Fat3 was shown to be expressed in glial cells and endothelial cells of the human brain^28^, however, in mouse, quantitative RNA-seq has revealed its neuronal expression to be ~6 times higher than in astrocytes and oligodendrocyte precursors^29^. The ratio between glial cells and neurons is, however, a factor likely to affect the level of Fat3 expression detected. This is of particular relevance to the brainstem, which has been recently described as an area with an extremely high ratio of non-neuronal cells to neurons^30^. In conclusion, given the complex interplay of expression levels in different cell types and the ratio of the different cell types, it is difficult to establish whether a complete knock out of *Fat3* has been obtained in brain-stem neurons. Nevertheless, we were unable to detect any alterations in the autophagy flux at global level also in this area (Fig. 5F–I) or any behavioural anomalies of these mice (Fig. 4). A single cell-level analysis may be necessary in this set up to establish whether this was due to incomplete recombination, unaffected glial autophagy masking any alterations in neuronal autophagy or lack of effect of *Fat3* knock-out on autophagy.

The importance of Fat cadherins in neurodegeneration is however strongly supported by the significant downregulation we report in DRPLA mouse models, which mirrors previous results obtained in *Drosophila* models and further highlights the special relevance of this regulation for the cerebellum. Importantly, robust down regulation of the *Fat* genes is observed at an early phenotypic stage, indicating that this downregulation may be causative of degenerating events and underly the subsequent, dramatic progression of cellular and behavioural defects in DRPLA mice^18^. The predominant expression of all mammalian *Fat* in the cerebellum indicate that they may play an essential role in neuronal homeostasis specifically in this brain area. It is yet unclear what level of overlap and eventual compensation there may be between the different Fat in the cerebellum, however, mutations in *FAT1* and *FAT2* have indeed been identified in a Dutch cohort of dominant ataxia patients^31^. *FAT2* is now recognised as the official cause for Spino-Cerebellar-Ataxia 45 (SCA45). Therefore, studying autophagic flux and neurodegeneration in *Fat1* deficient or *Fat2* deficient mice will be of high future interest in order to investigate the cellular mechanisms that underlie the role of those two Fat homologues in ataxia.

## Materials and Methods

### Animals and breeding

All experimental procedures with mice were carried out under a license from the Home Office according to the regulations set by the Animals Scientific Procedures Act 1986 (ASPA). This study was approved by the UK Home Office and King’s College London Animal Welfare and Ethical Review Body. The two DRPLA mouse strains C3; B6-Tg (Prnp-ATN1) 84Dbo/Mmmh (26Q) and C3; B6-Tg (Prnp-ATN1) 150Dbo/Mmmh (65Q)^17^ were recovered from the MMRRC repository and maintained by backcrossing to (CBA/Ca × C57BL/6J) F1 animals. Genotyping was performed as previosly described^18^. *Fat3*^*fl/fl*^ mice (Fat3^tm1.1Good^) were received from The Jackson Laboratory^26^. To generate neuron specific *Fat3* ko in postnatal mice, *Fat3*^*fl/fl*^ mice were cross bred with *Thy1Cre*(*ER*^*T2*^) transgenic mice (SLICK-H, received from the Jackson Laboratory). This SLICK-H line (Single-neuron Labelling with Inducible Cre-mediated KO) expresses both yellow fluorescent protein (YFP) and the CreER^T2^ fusion protein under the control of two different Thy1 promotors^23^. The B6.Cg-Gt(ROSA)26Sor < tm14(CAG-tdTomato)Hze>/J (gift from Fiona Watt) was used to assess *Thy1Cre*(*ER*^*T2*^) leakiness and efficiency. Breeding of *Fat3*^*fl/fl*^ mice with *Thy1Cre*(*ER*^*T2*^) transgenic mice generated a double transgenic line homozygous for *Fat3*^*fl*^ and hemizygous for *Thy1Cre*(*ER*^*T2*^) (*Fat3*^*fl/fl*^; *Thy1Cre*(*ER*^*T2*^)^*tg/*+^) after two generations. Both lines were backcrossed to C57.B6/J background for 6 generations prior to generation of double transgenic strains. In the offspring, tamoxifen at concentration of 20 mg/ml was injected at 4 weeks of age with doses of 75 mg tamoxifen/kg body weight on 5 consecutive days leading to a pan-neuronal Cre-mediated recombination. Control litter mates were also injected with tamoxifen and contained the *Fat3*^*fl*^ alleles but not the *Thy1Cre*(*ER*^*T2*^) transgene. In total, 8 mice were analyzed (n = 4 per group) in each experiment where control and KO mice were chosen from the same litter. To determine genotypes by PCR, DNA was extracted from ear biopsies. Primers: Fat3-LoxP-FW: 5′-TCAGCAGCCATACTGAGTGGTTCC-3′, Fat3-LoxP-REV: 5′-TTTCTGCACCCCTTCCCTAACAGTG-3′. Thy1-FW: 5′-TCTGAGTGGCAAAGGACCTTAGG-3′, Thy1-REV: 5′-CGCTGAACTTGTGGCCGTTTACG-3′. PCR conditions were 95 °C for 30 sec, 60 °C for 30 sec and 72 °C for 1 min for a total of 31 cycles.

### Behavioral analysis

For gait analysis, after 2 runs of training in the apparatus, front limbs and hindlimbs of mice were coated with blue and red ink, respectively. Subsequently, mice were put on a white sheet of paper (1 m long, 6 cm wide) and allowed to walk along the runway into an enclosed dark box. The test was performed once for each mouse. At least three subsequent steps were analysed for each limb where the first 10 cm of each run were excluded as the initiating movement. Representative footprints were scanned in and three parameters were analysed with ImageJ. (1) Stride length was determined as the distance between two subsequent steps. (2) front step width and (3) hind step width were measured as the distance between the connecting line of the proceeding and preceding step on the opposite side and the footprint to be measured. The mean of three values was used for subsequent analysis. Body weight and grip strength were assessed every week starting at five weeks of age. To assess grip strength, mice were guided along a wire-mesh grid attached to a grip strength monitor (Bioseb *In Vivo* Research Instruments) by holding them at the base of the tail. The maximum tension was recorded (g) by gently pulling the mouse away from the apparatus. The average of three trials per animal was subjected to statistical analysis.

### Western blot analysis

#### Brain dissections

Transgenic and control mice (four per genotype) were sacrificed by exposure to carbon dioxide gas in a rising concentration and subsequent dislocation of the neck. A stereomicroscope was used to dissect the brains in ice cold PBS. To separate the two hemispheres of the brain, medio-sagittal incision was performed and to detach the forebrain from the diencephalon, connecting fibres were cut. Cerebellar peduncles were disrupted to disassociate the cerebellum. The remaining part which contained diencephalon, midbrain, pons and formation reticularis are further referred to as brainstem. All tissues were deep frozen in 1.5 ml tubes in liquid nitrogen and stored at −80 °C.

#### Nuclear and cytoplasmic preparations

For protein extraction sequential lysis of the brain tissues was performed, resulting in fractions enriched in cytoplasmic/membranous and nuclear components. The left half of the cerebellum or brainstem were homogenized in RIPA buffer (137 mM NaCl, 20 mM Tris-HCl pH 7.5, 25 mM β-glycerophosphate, 2 mM EDTA, 1 mM sodium-orthovanadate, 1% (w/v) deoxycholate, 50 mM NaF, 1 supplemented with Complete protease inhibitor cocktail (Roche)) containing 1% (v/v) IGEPAL-630 with a blue plastic pestle that fit in a 1.5 ml Eppendorf tube. All samples were exposed three time to repeated freezing in liquid nitrogen and thawing on ice with occasional vortexing. Subsequently, samples were centrifuged at 4 °C for 15 min at full speed. The soluble fraction enriched in cytoplasmic proteins was removed and saved. The pellet consisting of the white upper layer and grey insoluble pellet was washed with RIPA buffer with 1% (v/v) TRITON X-100. The resulting pellet was dissolved in RIPA buffer with TRITON X-100 by sonification. All lysates were aliquoted, frozen in liquid nitrogen and stored at −80 °C.

#### SDS-PAGE and western blot assay

To determine protein concentration, a BCA assay kit (Thermo Scientific) was used with a microplate reader reading of absorbable at 562 nm. After protein lysates were denatured in Laemmli buffer for 5 min at 95 °C, 50 µg of supernatant protein and 25 µg of pellet protein were loaded on a 12% polyacrylamide gel. A discontinuous Laemmli SDS-PAGE was used with the Bio-Rad mini Protean 3 system and proteins were transferred onto nitrocellulose membrane (Enhanced chemiluminescence, ECL, 0.2 µm pore size, Amersham). To block unspecific binding, membranes were incubated in 5% (w/v) low-fat milk powder in Tris-buffered saline supplemented with 0.1% (v/v) tween (TBST). Primary antibodies were incubated in blocking solution at 4 °C overnight: rabbit α-LC3 (1:1000, MBL, PD014), mouse α-p62 (1:1000, Abnova, H00008878-M01), mouse α-Tubulin (1:10000, Sigma, T9026). HRP-conjugated α-mouse and α-rabbit secondary antibodies (Calbiochem) were applied for 1 hour at room temperature in blocking solution. SuperSignal West Pico Chemiluminescent Substrate (Thermo Scientific) and the ChemiDoc™ XRS+ System were used to visualize the protein of interest. Densiometric analysis was performed using the Image Studio Lite software.

### RNA extraction and qPCR

For the exploratory pretrial the brains of wild type and 65Q mice (n = 3) on the original C57BL/6J; C3H genetic background were dissected in ice cold PBS under stereo microscope. The olfactory bulb was separated by a coronal cut. The two forebrain hemispheres were separated by medio-sagittal incision starting at the thalamic level. The hippocampus was extracted by lifting the cortical layers laterally starting from the medial incision separating the two forebrain hemispheres. The striatum was dissected by stripping it free from the cortical layers by cutting along the fibers of the corpus callosum and separated from the thalamus by two inclined cuts. The cerebellum was separated by disruption of cerebellar peduncles. The remaining midbrain, pons and formatio reticularis (brainstem) was separated from the diencephalic portions including thalamus by a coronal cut.

For the confirmatory experiments, the cerebellum only was dissected from wild type, 26Q and 65Q mice (n = 6) all in the mixed (CBA/Ca × C57BL/6J) F1 background. The tissue was snap frozen in liquid nitrogen and stored at −80 °C. cDNA was generated using SuperScript III Reverse Transcriptase (Invitrogen). To quantify expression levels of *Fat* genes, cDNA template was amplified using Universal Probe Library-based (UPL library, Roche) qPCR in combination with TaqMan Universal PCR Master mix on an ABI 7900HT real-time PCR system (Applied Biosystems). All sequences used for the quantification are listed in Table 1.

**Table 1:** Primer and UPL probes for qRT-PCR.

Gene	UPL	Primer forward	Primer reverse
*Hprt1*	95	cctcctcagaccgcttttt	aacctggttcatcatcgctaa
*β-actin*	56	aaggccaaccgtgaaaagat	gtggtacgaccagaggcatac
*Fat1*	89	acgcggttgtcatgtacg	tgcagtcttccggtatgaatc
*Fat2*	89	aacttgtggctactctgaagacg	cagggggtctccctctgt
*Fat3*	56	ggggactctgtcattctgct	cccacaatggaaaaacgaat
*Fat4*	97	cacaaggcattcttgaccag	tgaccagaagtccacacagg

For *Fat3* ko mice, phenol-chloroform RNA extraction from mouse brains was performed according to the TRI Reagent® user guide (Sigma). cDNA was generated using the SuperScript III Reverse Transcriptase (Invitrogen). Each reaction initially contained 2.5 µg of RNA with a reaction volume of 20 µl. To quantify *Fat3* expression levels in neuronal *Fat3* ko mice, semi-quantitative PCR was performed. Therefore, cDNA was diluted 1:50 and PCR was performed. PCR conditions were 95 °C for 30 sec, 60 °C for 30 sec and 72 °C for 1 min for a total of 31 cycles. To prevent the amplification of genomic DNA, PCR primers were designed that bind to adjacent exons and span an intronic splicing site. Three different primer pairs were designed binding to exon 14/15, exon 21/22 and exon 23/24 of *Fat3*. In addition, primer pairs detecting *Fat1* and *Hprt1* were used as controls. Primer pairs are listed in Table 2. The PCR products were separated on a 10% polyacrylamide gel which was run in TAE buffer. For post-staining of DNA, the gel was incubated in EtBr in TAE buffer (1: 100 000) for 5 min and imaged with a UVP MultiDoc-It^TM^ imaging system. Densiometric analysis was performed using the Image Studio Lite software.

**Table 2:** Primer pairs used for semi-quantitative PCR.

Gen/transgene	Fw/Rev	Primer sequence 5′ → 3′
*Hprt1*	Fw	CCTCCTCAGACCGCTTTTT
	Rev	AACCTGGTTCATCATCGCTAA
*Fat1*	Fw	ACGCGGTTGTCATGTACG
	Rev	TGCAGTCTTCCGGTATGAATC
*Fat3* exon 14/15	Fw	GGGGACTCTGTCATTCTGCT
	Rev	CCCACAATGGAAAAACGAAT
*Fat3* exon 21/22	Fw	AATGTACACTCGGGCCAACC
	Rev	GAGTGCCAGCTCCCATCATT
*Fat3* exon 23/24	Fw	TCACAGCCTGCTTCCCTAAC
	Rev	CTTCTCGCTCACACTCGTCC

### Statistical anaysis

Statistical analysis was performed manually with the GraphPad Prism software. Both the Kolmogorov-Smirnov-test for normality and the F-test for equal variances were performed. After the data passed those tests, an unpaired Student’s t-test or one-way ANOVA was applied to test for statistically significant differences between KO and control group. Where data did not pass the normality test, the Mann-Whitney-test or Kruskal-Wallis test was performed. Different levels of significance were defined as following: ns for not significant, *p ≤ 0.05, **p ≤ 0.01 and ***p ≤ 0.001.
